# Axillary lymph node imaging in mRNA, vector-based, and mix-and-match COVID-19 vaccine recipients: ultrasound features

**DOI:** 10.1007/s00330-022-08846-9

**Published:** 2022-05-13

**Authors:** Alba Cristina Igual-Rouilleault, Ignacio Soriano, Arlette Elizalde, Paola Leonor Quan, Alejandro Fernandez-Montero, Carolina Sobrido, Luis Pina

**Affiliations:** 1 Department of Radiology, Clínica Universidad de Navarra, Avenida Pío XII, 36, 31008 Pamplona, Spain; 2 Breast Imaging Unit, Department of Radiology, Clínica Universidad de Navarra, Avenida Pío XII, 36, Pamplona, Spain; 3 Department of Allergy and Clinical Immunology, Clínica Universidad de Navarra, Avenida Pío XII, 36, Pamplona, Spain; 4 Prevention and Health Service, Clínica Universidad de Navarra, Avenida Pío XII, 36, Pamplona, Spain; 5 Breast Imaging Unit, Department of Radiology, Clínica Universidad de Navarra, C. Marquesado de Sta. Marta, 1, Madrid, Spain

**Keywords:** COVID-19 vaccines, Unilateral axillary lymphadenopathy, Ultrasound, Age, Follow-up

## Abstract

**Objectives:**

To assess ultrasound characteristics of ipsilateral axillary lymph nodes after two doses of four different COVID-19 vaccination protocols, to determine whether these parameters differed with age, and to describe how they changed on follow-up imaging.

**Methods:**

A total of 247 volunteer employees from our center who had received two doses of COVID-19 vaccination were recruited and followed prospectively. Axillary ultrasound of the ipsilateral vaccinated arm was performed the week after receiving the second dose to analyze lymph node features (number, long-axis, cortical thickness, morphology, and vascular imaging). Axillary lymphadenopathy resulting from four vaccination protocols—mRNA (BNT162b2, mRNA-1273), ChAdOx1-S, and mix-and-match—was compared. Analysis was conducted using the Kruskal-Wallis test and post hoc analysis with Bonferroni corrections. Nodal reactogenicity was evaluated for two age groups: young (< 45 years old) and middle-aged ( ≥ 45 years old). All parameters were compared between both groups using an unpaired-sample Student *t* test. A *p* value < 0.05 was considered statistically significant.

**Results:**

Significantly higher values for total number of visible nodes, cortical thickness, Bedi’s classification (*p* < 0.001), and vascularity (*p* < 0.05) were observed in mRNA vaccine recipients compared to full ChAdOx1-S protocol recipients. Moreover, mix-and-match protocol recipients showed greater nodal cortical thickness and higher Bedi’s classification than full ChAdOx1-S recipients (*p* < 0.001). Analyses between age groups revealed greater cortical thickness, Bedi’s classification, and color Doppler signal in younger patients (*p* < 0.05).

**Conclusions:**

Nodal parameters of Bedi’s classification and cortical thickness were more often increased in mRNA and mix-and-match vaccine recipients when compared to ChAdOx1-S vaccine alone, especially in younger patients.

**Key Points:**

• *Hyperplastic lymphadenopathy was observed more frequently in mRNA and mix-and-match vaccine protocols compared to full vector-based vaccination.*

• *Higher values for cortical thickness, Bedi’s classification, and color Doppler signal parameters were identified in younger patients.*

• *Observed lymph node findings normalized in greater than 80% of patients by the third month following vaccination.*

## Introduction

In December 2019, a new coronavirus (2019-nCoV or SARS-CoV-2) was identified in the Wuhan province (China). Human-to-human transmission spread the virus quickly to all countries on the globe and 3 months later, in March 2020, the novel respiratory disease caused by it (COVID-19) reached the status of global pandemic by the World Health Organization (WHO) [1]. Since then, the rollout of vaccination programs remains the most helpful action to contain coronavirus dissemination. Several COVID-19 vaccines have been authorized by the European Commission, via the European Medicines Agency, including novel mRNA (Pfizer-BioNTech BNT162b2, Moderna’s mRNA-1273) and viral vector-based (AstraZeneca ChAdOx1, Janssen Pharmaceuticals) vaccines [2, 3]. Additionally, in June 2021, the WHO’s Strategic Advisory Group of Experts on Vaccines approved the use of the Pfizer vaccine as a second dose after an initial dose of AstraZeneca [4], resulting in the so-called mix-and-match COVID-19 vaccine protocols.

Recent literature reports local and systemic symptoms of a predominantly mild severity in vaccine recipients, including pain at the injection site, fever, fatigue, and headache [5–7]. In a lesser percentage of patients, acute and delayed type hypersensitivity adverse events have also been described [8]. Moreover, widespread cases of axillary and supraclavicular adenopathy ipsilateral to the injection site have also been documented [9–15].

Occasionally, other vaccines (e.g., H1N1, papillomavirus, smallpox, measles, anthrax, and Bacille Calmette-Guerin) can induce reactive lymphadenopathy [16–20]. However, the appearance of hyperplastic lymph nodes after COVID-19 vaccination has become a particularly prevalent phenomenon, showing a high incidence and frequently alarming nodal features on imaging tests. The preferred imaging technique for their assessment is ultrasound (US) examination [21] due to its simplicity, possibility for real-time evaluation, good visualization of soft tissues, and lack of ionizing radiation.

With the rollout of COVID-19 vaccination campaigns on a population scale and the lack of information about these new vaccines, the aim of our study was to compare the US characteristics of axillary nodes ipsilateral to the injection site for four different vaccine protocols—mRNA (Pfizer, Moderna), viral vector-based, and mix-and-match COVID-19 vaccines, and to assess the influence of age in nodal reactogenicity.

## Materials and methods

### Study design

We conducted a prospective observational single-center study with the approval of the regional ethics committee and written informed consent of all participants.

Between February and July 2021, 512 employees from our center were invited to participate in this prospective study after receiving two doses of COVID-19 vaccination with either Pfizer, Moderna, AstraZeneca, or a mix-and-match COVID-19 vaccine protocol combining AstraZeneca with a second dose of Pfizer. According to our national center for disease, mRNA vaccines (Pfizer, Moderna) were provided to high-risk health care professionals (including doctors, nurses, physiotherapists, and others providing direct patient care) whereas vector-based vaccine (AstraZeneca) was offered to low-risk workers (administrative and social care professionals). Subjects who received AstraZeneca as a first dose of COVID-19 vaccination could choose either the mRNA vaccine (Pfizer), as our Ministry of Health recommended, or another dose of AstraZeneca for their second dose. Patients were included if they received two doses of any of these four COVID-19 vaccine protocols and if they were injected with both doses in the same arm. Patients with known onco-hematologic disease were excluded from the study. Finally, a total of 247 volunteers were recruited and followed prospectively with a monthly ultrasound follow-up examination. The vaccine or vaccines administered were recorded for all of them, as well as the clinical information (age and sex).

### Ultrasound acquisition

Ultrasounds were obtained using two different broad-band linear transducers with a band frequency of 8–13 MHz (Logiq E9, GE Healthcare; and Aplio i800 series ultrasound system, Canon Medical Systems Corporation). Participants were scanned the week after receiving the second vaccine injection. US examination was obtained from the ipsilateral axillary region of the vaccinated upper extremity.

Two third-year residents and two radiologists, with more than 20 years of experience in breast imaging, performed axillary US scans. The four radiologists were not blinded to the type of administered vaccine when initial US examination was performed. However, each patient’s ultrasound scan images were reviewed by the two expert radiologists without information about the type of administered vaccine.

### Data assessment

Multiparametric assessment of post-vaccine lymph nodes on ultrasound included total number of visible nodes in level I, maximum measurements of long-axis size and cortical thickness, morphological Bedi’s classification, and color Doppler evaluation. Morphological classification of axillary lymph nodes was conducted according to types 1–6 of Bedi’s cortical classification [22]. Finally, Doppler evaluation was performed using a four-degree scale employed in our institution: degree 0, no Doppler signal; degree 1, only hilar Doppler signal; degree 2, mild-moderate positive Doppler signal in hilar and cortical regions; degree 3, high positive Doppler signal in hilar and cortical regions. Maximum values of each variable were registered selecting different nodes if necessary (i.e., the cortical thickness and Bedi´s classification could be measured in one node, while the larger diameter or the color Doppler could be measured from a different one).

### Statistical analysis

All statistical analyses were performed in IBM SPSS Statistics software version 21.0. Distribution normality was tested using the Kolmogorov-Smirnov test. Differences in nodal US features according to different COVID-19 vaccine protocols were evaluated using the Kruskal-Wallis test and post hoc analysis with Bonferroni corrections. Quantitative continuous variables (number of nodes, long-axis size, and cortical thickness) and ordinal variables (Bedi’s classification and Doppler scale) were presented as median and interquartile range (IQR) due to non-normal distribution of all data. Assessment of demographic factors—age and sex—between mRNA (Pfizer, Moderna) and AstraZeneca vaccine recipients was performed using descriptive statistics. Additionally, the Mann-Whitney *U* test was conducted to compare median age between both sexes and to analyze if cortical thickness values differ between women and men.

Finally, to compare hyperplasic axillary lymph node reaction between different age groups, we classified mRNA and AstraZeneca vaccine recipients into two groups using 45 years as a cutoff point, based on the mean age of the sample and the median age of our country population. All US parameters were evaluated between both age groups using an unpaired-sample Student *t* test. In this analysis, quantitative continuous variables (number of nodes, long-axis size, and cortical thickness) were presented as mean ± standard deviation (SD) because of their normal distribution; and ordinal variables (Bedi’s classification and Doppler scale) were reported as median (middle value) ± interquartile range. For all comparisons, a *p* value < 0.05 was considered to be statistically significant.

## Results

### Patient characteristics

Out of 512 invited employees who fulfilled the inclusion criteria, 247 patients accepted to participate in this prospective study. Demographic data analysis (*n* = 247) showed an average age of 44.8 ± 12.1 (range 20–67) and a large percentage of women (85%). According to the COVID-19 vaccine protocols administered, subjects were divided into four groups: recipients of the Pfizer (91; 36.8%), Moderna (55; 22.3%), AstraZeneca (77; 31.2%), or mix-and-match COVID vaccination (24; 9.7%) (Table 1). No significant differences were seen when comparing the mean age of patients receiving mRNA (Pfizer, Moderna) and AstraZeneca vaccines (*p* = 0.685).

**Table 1:** Details of sample

Vaccine protocol	Vaccines administered (1st–2nd doses)	Total number of recipients (percentage)	Age years old (mean ± SD)
1	Pfizer-Pfizer	91 (36.8%)	43.77 ± 12.75
2	Moderna-Moderna	55 (22.3%)	47.67 ± 10.24
3	AstraZeneca-AstraZeneca	77 (31.2%)	45.94 ± 12.22
4	AstraZeneca-Pfizer	24 (9.7%)	39.33 ± 11.69

### Differences found in ultrasound parameters of axillary lymph nodes according to vaccine protocols, age, and sex

**Fig. 1:**
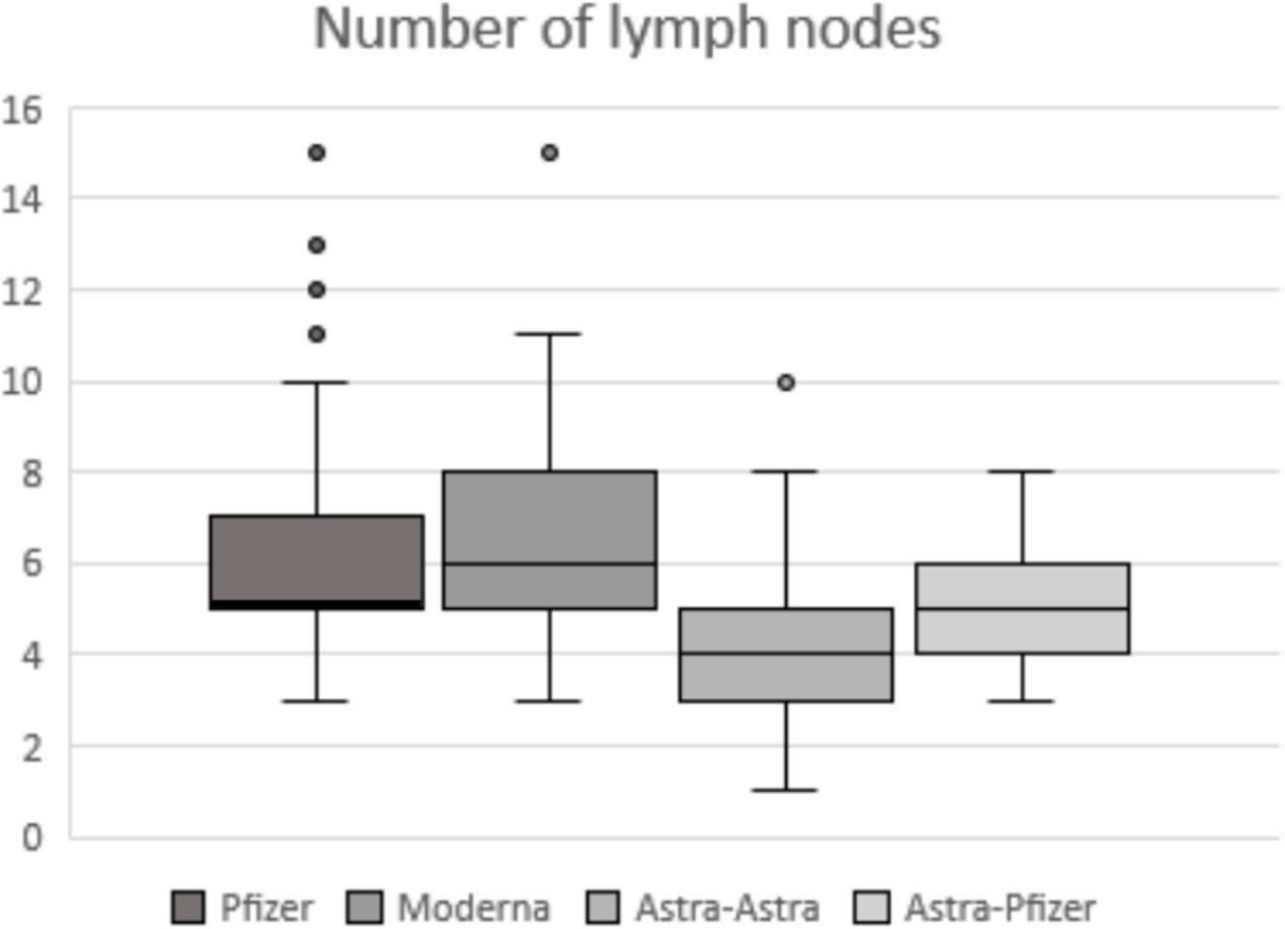
Box-whisker plot of lymph nodes’ number comparing quartiles across the fourth different vaccine protocols. Median (IQR) differs significantly between mRNA vaccines and full AstraZeneca vaccination: Pfizer and Moderna (5 (2); 6 (3), respectively) vs AstraZeneca (4 (2)) with post hoc analysis *p* value < 0.001

**Fig. 2:**
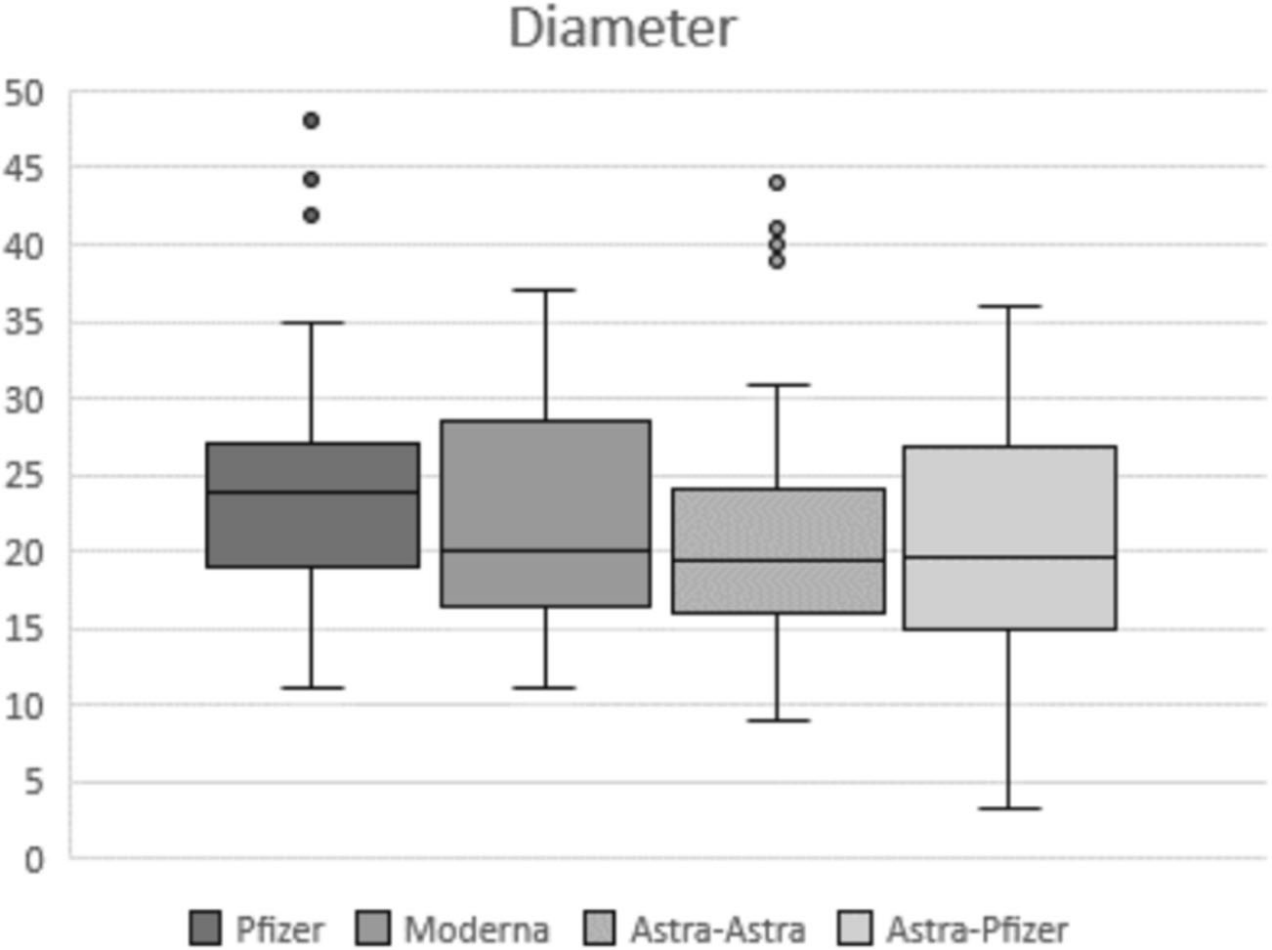
Box-whisker plot of diameter measurement comparing quartiles across the fourth different vaccine protocols. Median (IQR) differs significantly between complete Pfizer vaccination and full AstraZeneca vaccine protocol (23.9 (8) vs 19.3 (8), post hoc analysis *p* = 0.002)

**Fig. 3:**
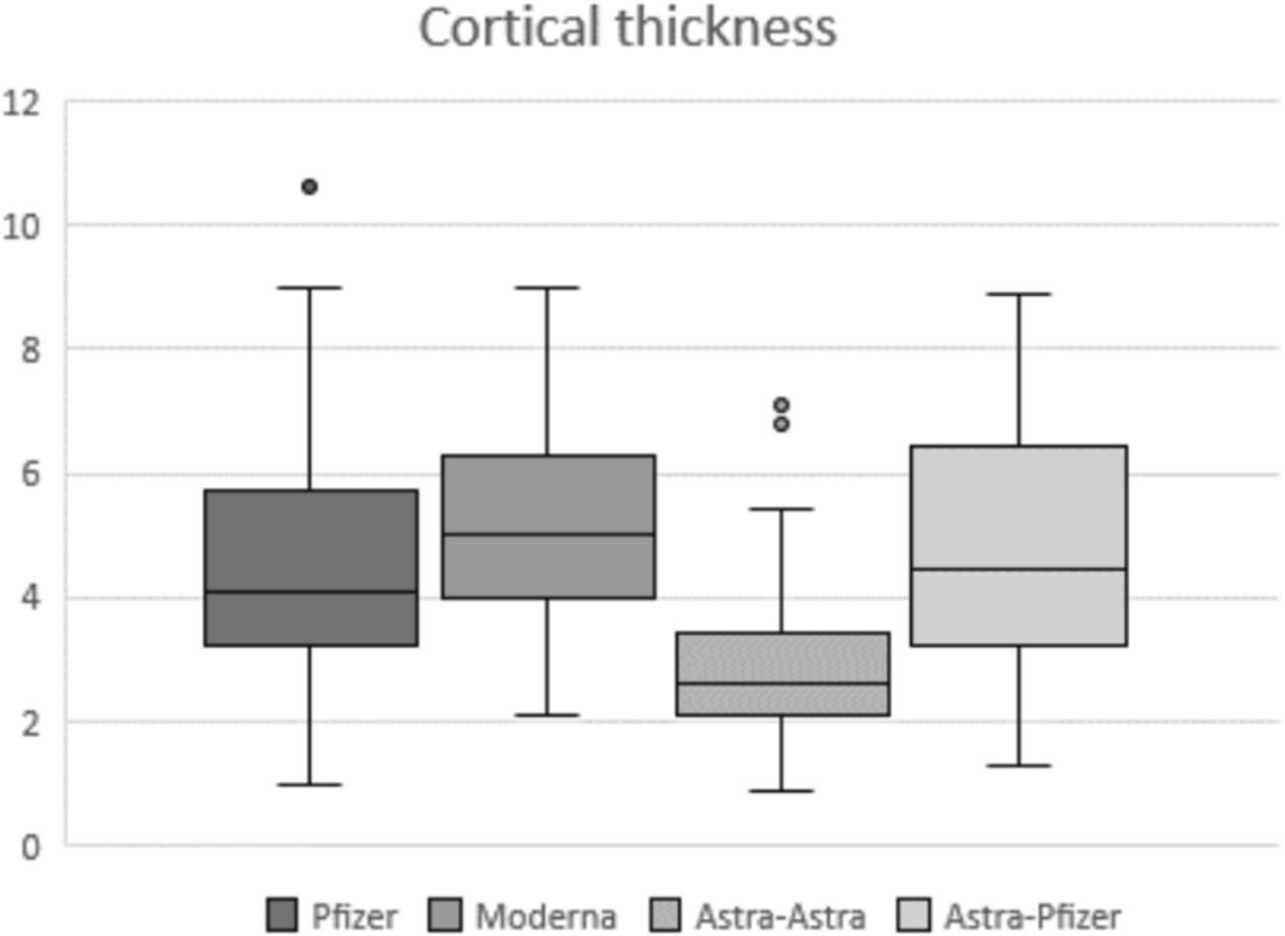
Box-whisker plot of cortical thickness comparing quartiles across the fourth different vaccine protocols. Median differs significantly between mRNA and mix-and-match COVID vaccinations compared to two doses of AstraZeneca with significantly greater cortical thickness (post hoc analysis *p* < 0.001)

**Fig. 4:**
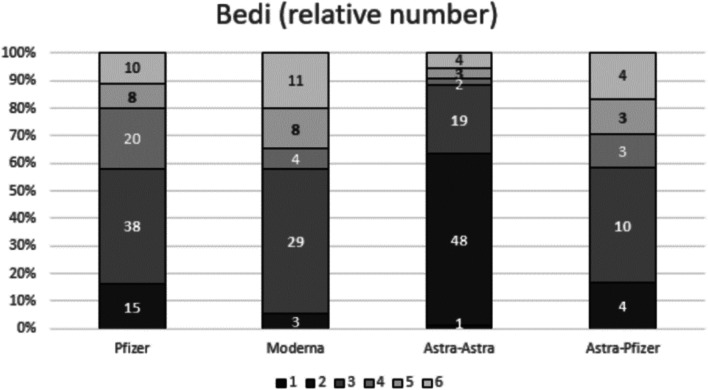
Sample distribution and comparison per Bedi’s classification grades (number of patients indicated for each grade) between the four different vaccine protocols. Patients with mRNA or mix-and-match COVID vaccination presented significantly higher morphological grade than those who received two doses of AstraZeneca (post hoc analysis *p* < 0.001). Larger percentage of lymph nodes categorized as grades 1 and 2 was observed in full AstraZeneca vaccine recipients (63.6%) compared to the other three vaccine protocols: Pfizer (16.5%), Moderna (5.5%), AstraZeneca and Pfizer (16.7%)

**Fig. 5:**
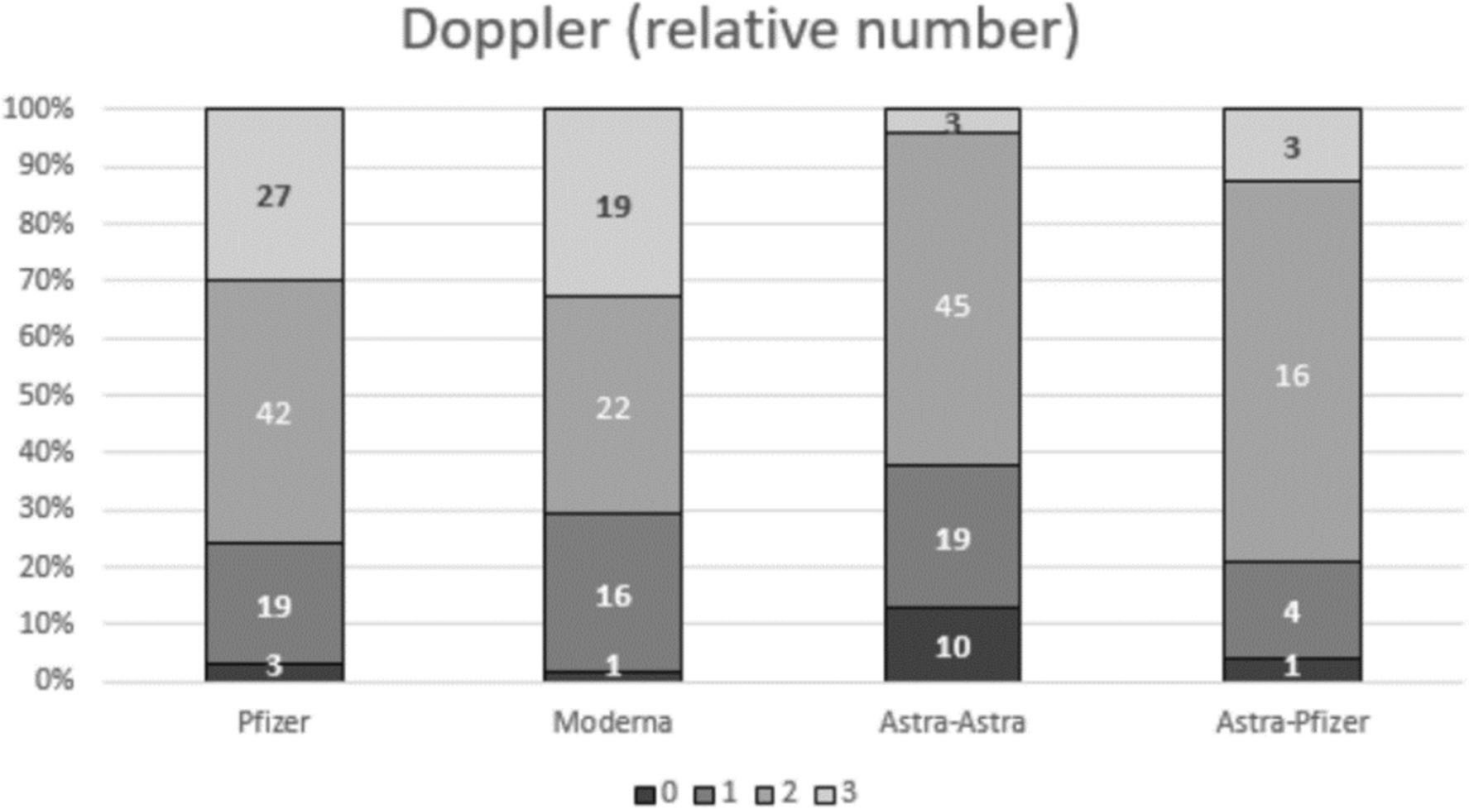
Sample distribution and comparison per Doppler scale degrees (number of patients indicated for each grade) between the four different vaccine protocols. Patients fully vaccinated with Pfizer and Moderna showed more significantly

Statistical analysis considering the four different vaccine groups showed statistical significant differences in the pairwise comparison of each nodal parameter assessed. Regarding the total median (IQR) number of total visible ipsilateral axillary lymph nodes, patients vaccinated with two doses of mRNA vaccine showed a significantly larger number of level I axillary nodes when compared to patients with AstraZeneca complete vaccination. Recipients who received Pfizer or Moderna mRNA vaccines demonstrated 5 (2) or 6 (3) nodes respectively vs AstraZeneca recipients who demonstrated 4 (2) nodes (Fig. 1) with *p* < 0.001 in both post hoc analyses. In terms of the maximum measurement of node long-axis, full Pfizer vaccine recipients presented a significantly longer median (IQR) nodal diameter than those who received both doses of AstraZeneca (23.9 (8) vs 19.3 (8), post hoc analysis *p* = 0.002) (Fig. 2). For the cortical thickness measurement and the corresponding grade of Bedi’s classification, patients with complete mRNA vaccination and those who received mix-and-match COVID vaccination presented significantly greater cortical thickness and higher grade of Bedi’s classification than patients with two doses of AstraZeneca (post hoc analysis *p* < 0.001). Assessment of morphological Bedi classification demonstrated a larger percentage of lymph nodes categorized as grades 1 and 2 in full AstraZeneca vaccine recipients (63.6%) compared to the other three vaccine protocols: Pfizer (16.5%), Moderna (5.5%), AstraZeneca and Pfizer (16.7%) (Figs. 3 and 4). Concerning the color Doppler scale, patients fully vaccinated with Pfizer and Moderna showed a more significantly elevated Doppler signal than patients fully vaccinated with AstraZeneca (post hoc analysis *p* value = 0.001 and *p* = 0.035, respectively) (Fig. 5).

Comparative analysis of the lymph node response between young (104 subjects, 42.1%) and middle-aged patients (143 subjects, 57.9%) demonstrated more significant cortical thickness, higher grade in Bedi’s classification, and more intense color Doppler signal in younger patients. Differences in lymph node number and diameter (when assessing on the basis of longest lymph node diameter irrespective of lymph node morphology) were found, but were not statistical significant (Table 2).

**Table 2:** Mean (± SD) and median (± IQR) value comparison of quantitative and ordinal variables, respectively, between the two age groups of patients: young (< 45 years old) and middle-age (≥ 45 years old) patients

	< 45 years old	≥ 45 years old	*p* value
Number of lymph nodes (mean ± SD)	5.70 ± 2.33	5.52 ± 2.29	0.551
Diameter (mm, mean ± SD)	22.39 ± 6.78	22.24 ± 7.32	0.866
Cortical thickness (mm, mean ± SD)	4.69 ± 1.99	3.87 ± 1.75	0.001
Bedi’s classification (median ± IQR)	3 ± 2	3 ± 2	0.006
Doppler signal (median ± IQR)	2 ± 1	2 ± 1	0.005

The median age comparison between women and men showed statistically significant differences between women (45 ± 12) and men (40 ± 14) with *p* = 0.034. Regarding cortical thickness comparison between both sexes, no statistically significant difference was observed between women (4.1 ± 1.8) and men (4.7 ± 2) with a *p* value of 0.063. Finally, a supplementary US follow-up examination was offered to mRNA vaccine recipients who presented with a cortical thickness greater than 3 mm (125). The follow-up was available in 60.3% of patients (67/125). Out of these patients, 25 (37.3%) achieved normalization after 1 month, 20 (29.9%) after 2 months, 11 (16.4%) after 3 months, 2 (3%) after 4 months, and 2 (3%) after 5 months, and 7 (10.4%) continued presenting a cortical thickness greater than 3 mm beyond the fifth month after the second vaccination dose.

## Discussion

**Fig. 6:**
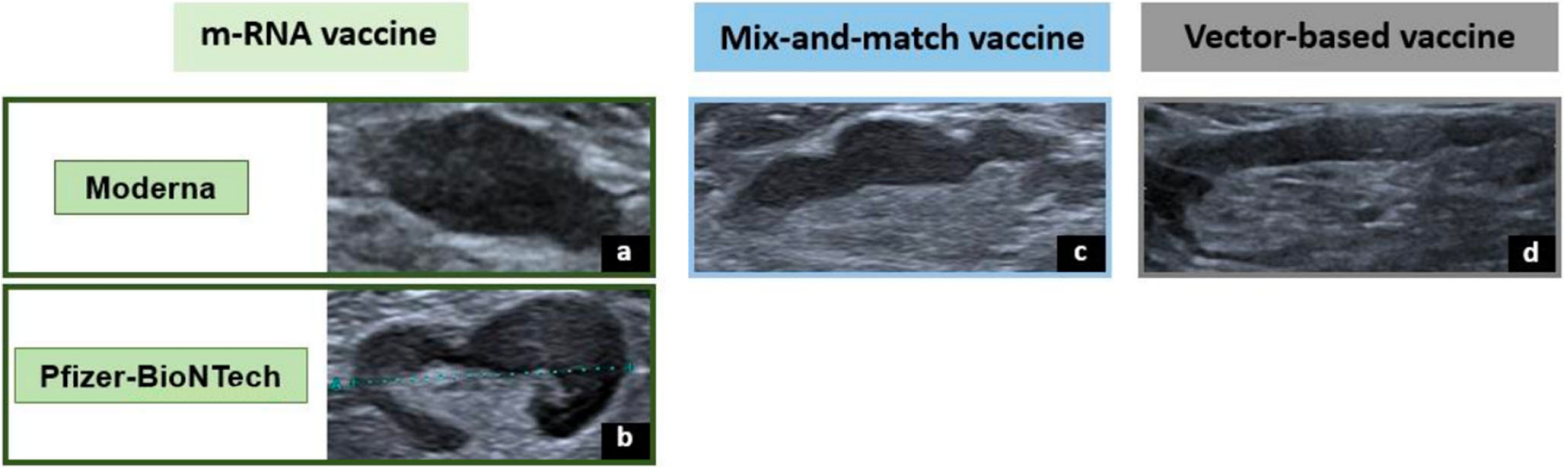
Comparative lymph node morphology in imaging ultrasound from vaccine recipients with different vaccination protocols. **a** A type Bedi 6 node with an absent hilum seen in a volunteer vaccinated with Moderna protocol. **b** A type Bedi 5 node with a focal hypoechoic cortical lobulation detected in a volunteer vaccinated with Pfizer protocol. **c** A type Bedi 4 node with a generalized lobulated cortex seen in a volunteer after receiving the mix-and-match protocol. **d** A type Bedi 3 node with a diffuse regular hypoechoic cortex greater than 3 mm in a volunteer vaccinated with full AstraZeneca protocol

Nodal reactogenicity induced by COVID-19 vaccinations has been reported in recent literature as the clinical pandemic era conundrum [23]. To approach this new diagnostic dilemma, our study highlights the influence of the vaccine protocol administered with more frequently hyperplastic lymphadenopathy in mRNA and mix-and-match vaccine protocols compared to full vector-based vaccination (Fig. 6); and the effect of age with higher values for cortical thickness, Bedi’s classification, and color Doppler study in younger patients. Moreover, we observed normalization of lymph node findings in greater than 80% of patients by the third month following vaccination.

Ultrasound morphologic features were assessed in nodes located below the lower edge of the pectoralis minor muscle (level I) because it is the region where palpable axillary lymphadenopathy was most frequently reported after vaccination. In all volunteers, ultrasound examination was performed studying each visible node located in that axillary station. The objective was to obtain a global vision of the lymphatic drainage in the vaccinated arm, to evaluate the influence of age and gender, and to collect the maximum value for each nodal parameter assessed, thus allowing for greater reproducibility. For this reason, the most suspicious lymph node per Bedi classification with the higher cortical thickness was sometimes not chosen as the suspicious by largest diameter or by color Doppler assessment. We categorized reactive lymph nodes following the Bedi morphologic classification (normally proposed as a way to objectively categorize the appearance of pathologic lymph nodes) because cortical thickness secondary to tumor cell deposit may be indistinguishable in ultrasound to one secondary to cortical sinuses expansion in nodal inflammation.

In our prospective cohort, all vaccinated volunteers (247) were healthy individuals with no reason to develop lymph node enlargement. Due to the lack of malignant risk factors, axillary reactive nodes detected ipsilateral to the vaccinated arm were regarded as physiologic post-vaccine lymphadenitis. Women represented the largest percentage of the sample (85%) due to a higher proportion of female employees in the health care and social sector [24]. The mean age of recruited patients was similar to the mean age of population in our country (43.8 years old) [25], so according to this reported demographic data, we used a cutoff of 45 years to classify all mRNA and AstraZeneca vaccine recipients (*n* = 223) into two age groups: young (< 45 years old) and middle-aged (≥ 45 years old) patients. Before studying the possible effect of age in nodal reactogenicity, we previously ruled out the possibility of an age bias among the recipients of the four different COVID-19 vaccine protocols. Data analysis confirmed no significant differences comparing the mean age of patients receiving mRNA (Pfizer, Moderna) and AstraZeneca vaccines and comparison between these two different age groups demonstrated a more significant lymph node response in younger patients compared to the middle-aged group, showing greater cortical thickness, Bedi’s grade classification, and color Doppler signal. The findings of decreased responsiveness in patients within the older age group may be attributed to the immunosenescence process. This age-related “state” of immune loss of function is characterized by a hindered capacity to launch effective humoral and cellular responses, with detriment of action against pathogens and deficiency of the immune response to vaccines [26, 27]. Our results suggest that this well-known decline of the physiological immune systems could manifest earlier than previously described in literature. In fact, the *p* value near the limit to judge significant the difference between cortical thickness values between women and men can be attributed to the statistical difference in median age with older women and, consequently, decreased responsiveness in that group.

Even if strong immune responses elicited by COVID-19 vaccinations have already been reported by the scientific community, our study demonstrated a more intense lymph node reaction in mRNA vaccine recipients when compared to AstraZeneca ones. Specifically, lymphadenopathy evoked by the Pfizer vaccine showed superior values in all US parameters assessed, and lymphadenopathy associated to the Moderna vaccine demonstrated similar differences, except for the maximum long-axis size of lymph nodes (not statistically significant results for this variable). These findings are in contrast to a previous study carried out by Cocco et al (2021), describing lymphadenopathy after COVID-19 vaccination with Pfizer, Moderna, and AstraZeneca vaccines in 24 patients, which reported no significant differences when comparing the US features of lymphadenopathies between the three COVID-19 vaccines [28]. This may be due to a small sample size employed and the consequent limitation of the study in detecting differences. We believe the larger sample size of our study, which was ten times greater, improved statistical power of our research with more consistent results.

Moreover, our results demonstrated that a mix-and-match COVID-19 vaccination protocol induced greater cortical thickness of axillary lymph nodes and higher grade of Bedi’s classification when compared to AstraZeneca full vaccination. The larger percentage of lymph nodes with normal appearance in Bedi’s classification (grades 1 and 2) was identified in AstraZeneca recipients with almost 65% of volunteers receiving that vaccine protocol. These findings support previously published results on higher nodal reactogenicity induced by mRNA vaccines [29]. All COVID-19 vaccine platforms are administered intramuscularly, but their mechanism of action differs. The mRNA vaccines (Pfizer and Moderna) use the virus’s genetic material—RNA—to prompt the body to create antibodies, while other vaccines rely on viral vectors (AstraZeneca), or modified versions of a different virus, to prompt an immune response [30]. Plausible explanations for the differences found in lymphadenopathy responses may be linked to these distinct mechanisms between vaccine groups. The mRNA component is a particle known to be recognized by a vast number of cell surface, and endosomal and cytosolic immune receptors [31], and great efforts are made to ensure proper purification of mRNA-containing products in order to avoid unwanted innate immune activation [32]. This intrinsic capability of inducing immune responses could explain the differences in nodal reactogenicity between vaccine types.

Challenges faced by radiologists when diagnosing patients seeking medical care after COVID-19 vaccination are highly dependent on patient context because post-vaccine lymphadenopathy can mimic nodal disease. The results of our study conclude that hyperplastic lymphadenopathy seen in healthy patients with no oncologic history, within 3 months of vaccination, does not require to perform the differential diagnosis with malignant lymph nodes. However, our results suggest as possible alarm signs for a pathologic process the identification of lymph nodes classified as Bedi types 4, 5, and 6 in middle-aged (≥ 45 years old) vector-based vaccine recipients, especially after 3 months. Our findings largely support this conclusion since normalization of cortical appearance occurred by the third month after vaccination in more than 80% of the patients who underwent follow-up in this study.

The findings in this report are subject to some limitations. Firstly, the absence of US examination of other nodal locations (i.e., subclavian, submandibular) means we do not have comparative information in terms of regional changes outside of the studied zone. Secondly, the small number of volunteers receiving the mix-and-match vaccine rendered this particular subclass quantitatively distinct from the others. Thirdly, imaging scans were obtained by two different US equipment which could induce correlation biases. Furthermore, the selection of health care workers as the target population eliminated the possibility to report nodal feature changes in extreme ages (sample selection bias). Additionally, follow-up axillary ultrasound was limited to mRNA vaccine recipients and long-term follow-up data past 5 months was not available. However, from our results showing the time to normalization of cortical thickness in mRNA vaccine recipients, it could be inferred that vector-based mix-and-match vaccine recipients would demonstrate earlier normalization of lymph node appearance given that these two vaccine protocols resulted in decreased cortical thickness and lower grade Bedi classifications compared to full two-dose mRNA vaccination. Moreover, the lack of a baseline axillary ultrasound examination of vaccine recipients prior to vaccination could be considered a limitation of this study. However, our approach was to study axillary lymph node appearance in a healthy population with no known reason for axillary adenopathy and who would not routinely undergo axillary ultrasound imaging. Lastly, a final limitation of the study could be the lack of biopsy pathology data for lymph nodes showing persistent cortical thickness more than 5 months post vaccination.

In conclusion, mRNA vaccines induced a more significant increase in axillary lymph node parameters, especially in younger individuals. The known particular reactogenicity of novel mRNA vaccines and the improved scientific solidity of these findings provide new insights to previous publications. In particular, our study offers a greater understanding of expected normalization of lymph node appearance over time. Because lymph node reactogenicity was greater in younger patients and because > 80% of lymph nodes normalized in appearance by 3 months after vaccination, older patients with an abnormal appearance of axillary nodes more than 3 months following vaccination should raise concern for other pathology and consideration for biopsy.

## Abbreviations

2019-nCOV: 2019 novel coronavirus
COVID-19: Coronavirus disease 2019
EUSOBI: European Society of Breast Imaging
mRNA: Messenger ribonucleic acid
SARS-CoV-2: Severe Acute Respiratory Syndrome Coronavirus 2
US: Ultrasound
WHO: World Health Organization

## Declarations

### Guarantor

The scientific guarantor of this publication is Dr. Pina.

### Statistics and biometry

One of the authors has significant statistical expertise.

### Study subjects or cohorts overlap

A recent publication has sought to describe the influence of the COVID-19 vaccine on ipsilateral axillary lymph node features, comparing their nodal basal features with their characteristics after the first and second vaccination dose (Igual-Rouilleault AC, Soriano I, Quan PL, Fernández-Montero A, Elizalde A, Pina L (2021) Unilateral axillary adenopathy induced by COVID-19 vaccine: US follow-up evaluation. Eur Radiol. DOI: 10.1007/s00330-021-08309-7). The 91 patients included in the cohort of this publication have also been included in this new research representing approximately 37% of the sample. However, in this project, our objective was to assess ultrasound characteristics of ipsilateral axillary lymph nodes after two doses of four different COVID-19 vaccination protocols—mRNA (Pfizer, Moderna), AstraZeneca, and mix-and-match (AstraZeneca and Pfizer)—and to determine also whether these parameters differed with age and how they changed on follow-up imaging.

### Methodology

• prospective

• observational study

• performed at one institution
